# Individualized electrode subset improves the calibration accuracy of an EEG P300-design brain-computer interface for people with severe cerebral palsy

**DOI:** 10.3389/fnhum.2026.1720969

**Published:** 2026-03-26

**Authors:** Si Long Jenny Tou, Seth A. Warschausky, Petra Karlsson, Jane E. Huggins

**Affiliations:** 1 Direct Brain Interface Laboratory, University of Michigan, Ann Arbor, MI, United States; 2 Institute of Biomedical Engineering, University of Toronto, Toronto, ON, Canada; 3 Department of Physical Medicine and Rehabilitation, University of Michigan, Ann Arbor, MI, United States; 4 Cerebral Palsy Alliance, University of Sydney, Sydney, NSW, Australia; 5 Department of Biomedical Engineering, University of Michigan, Ann Arbor, MI, United States; 6 Neuroscience Graduate Program, University of Michigan, Ann Arbor, MI, United States

**Keywords:** brain-computer interface, customization, developmental disabilities, EEG, P300

## Abstract

**Introduction:**

This study examined the effect of individualized electroencephalogram (EEG) electrode location selection for non-invasive P300-design brain-computer interfaces (BCIs) in people with varying severity of cerebral palsy (CP) in a post-hoc offline analysis.

**Methods:**

A forward selection algorithm was used to select the best performing eight electrodes (of an available 32) to construct an individualized electrode subset for each participant. Custom electrode subset size was chosen to be 8 because BCI accuracy of the individualized subset was compared to accuracy of a widely used default subset.

**Results:**

Across 51 participants, individualized subsets improved calibration accuracy only for the severe CP cohort (mean +28.6% absolute; 95% CI [13.4%, 46.1%]; *p* < 0.0001). No group-level benefit was detected for mild CP or typically developing controls, although several individuals in these groups improved (2/17 mild CP; 1/10 controls). In the subset with held-out testing data (mild CP and controls), calibration gains did not translate to higher testing accuracy; among controls, the subset effect was reduced on testing (−9.6%, 95% CI [−13.3%, −5.8%], *p* < 0.0001), with no evidence of change for mild CP. Participants with severe CP typically required larger subsets to approach asymptotic accuracy, whereas ≤ 8 electrodes were sufficient for most others.

**Discussion:**

The findings suggested that electrode selection can accommodate atypical neuroanatomy in people with severe CP, while the default electrode locations are sufficient for people with milder impairments from CP and typically developing individuals.

## 1 Background

A P300-design electroencephalogram (EEG) brain-computer interface (BCI) interprets brain activity to translate users' intentions into computer commands to type or generate speech for communication without physical movement (Farwell and Donchin, 1988). The P300 is a positive in magnitude component occurring about 300 ms after perception of an expected target stimulus that occurs infrequently and randomly. However, the P300 BCI design does not specifically check for P300s. Instead it will detect any event-related potential (ERP) components including the P300, N200, etc. (Kaufmann et al., 2011). The EEG P300-design BCI was first proposed in Farwell and Donchin (1988) with only one electrode location used (Farwell and Donchin, 1988). Subsequent studies using additional electrodes showed improved BCI performance (Sellers and Donchin, 2006; Krusienski et al., 2008), and recent P300-design BCIs usually collect data from a set of multiple electrodes with fixed default locations. Some of the latest P300-design BCI studies use up to 64 electrodes (Krusienski et al., 2008; Bittencourt-Villalpando and Maurits, 2018). A larger electrode set, however, increases cost and setup time, and makes the BCI less practical for end users (Bhandari et al., 2024; Colwell et al., 2014; Yu et al., 2015; Cecotti et al., 2011). This is particularly true when considering home use of a BCI in which a caregiver must set up the BCI for the user. Indeed, only 65% of potential end users with spinal cord injury would be satisfied with 10-20 minutes of setup time (Huggins et al., 2015). In addition, increasing the size of an electrode subset may only yield minimal improvement in accuracy beyond a certain point (McCann et al., 2015; Bhandari et al., 2024). Thus, for cost and setup time considerations, in-home BCI studies have used only 8 electrodes (Holz et al., 2015; Wolpaw et al., 2018).

BCI research often focuses on improving the classifiers that interpret the brain activity. Some commonly used classifiers, such as stepwise linear discriminant analysis (SWLDA), incorporate feature selection as part of the training process. However, this feature selection is performed across all extracted features irrespective of their electrode of origin, and therefore does not reduce the number of electrodes required for data acquisition. As a result, such approaches offer limited benefit for reducing hardware cost or setup time. Similarly, neural network-based approaches can integrate information across multiple electrode locations to learn latent representations and have demonstrated notable gains in classification accuracy (Wang et al., 2021, 2020; Ji et al., 2025). Nevertheless, these methods typically rely on high-density electrode montages and thus do not address the practical challenges associated with electrode count, including setup burden and accessibility for end users. If cost and setup time lead to reduction of the number of electrodes, it is important to understand if the default electrode subsets used by in-home studies (Holz et al., 2015; Wolpaw et al., 2018) are indeed appropriate for all users.

Studies have shown that P300-design BCIs perform better when used by typically developing participants compared to those with neurological conditions (Halder et al., 2015; Piccione et al., 2006). The mechanisms underlying these differences have not been clearly identified. However, this disparity may stem in part from the effects of underlying differences in neuroanatomy or brain function directly related to the neurological diagnosis. This raises concerns that commercialization efforts that seek to minimize the number of electrodes will result in BCIs that do not work for people with physical impairments. If electrode selection can create individualized electrode subsets that provide better signal detection, while using a minimal number of electrodes, BCIs can become more accurate, lower in cost, and faster to set up. Using an effective electrode selection algorithm to obtain custom electrode subsets has the potential to remove barriers to BCI use outside the laboratory environment.

Many electrode selection methods are discussed in the literature on EEG-BCI, including filtering, wrapping, embedded, and hybrid electrode selection techniques for EEG (Alotaiby et al., 2015; Bhandari et al., 2024). Filtering techniques use independent criteria to assess the usefulness of electrodes. Wrapper techniques, on the other hand, depend on a classification algorithm or machine learning to evaluate an individual's electrode subset. While filtering techniques are computationally much cheaper and faster than wrapper techniques, often they are limited by low accuracy (Alotaiby et al., 2015; Bhandari et al., 2024). Examples of filtering techniques include minimizing within-class variance, maximizing between-class variance, maximizing entropy, and using common spatial pattern filter coefficients. Wrapper techniques include support vector machine (SVM) to rank the contributions of each selected electrode, and regularized linear discriminant classifier (RLDC) (Alotaiby et al., 2015; Bhandari et al., 2024). Embedded techniques incorporate electrode selection into the construction of the classifier. Hybrid is a combination of the above techniques (Alotaiby et al., 2015). Other methods include using principal component analysis (PCA) to assess contributions of each electrode (Yildiz and Bergil, 2015), inconsistencies of classifiers (Yang et al., 2014), and backtracking search optimization (Dai and Wei, 2017).

Electrode selection wrapper methods that have been evaluated with P300-design BCI data range from deterministic searches, such as exhaustive search (Colwell et al., 2014), forward or backward (including n-forward-m-reverse and jumpwise) selection (Colwell et al., 2014; McCann et al., 2015), to probabilistic and machine-learning approaches such as sparse Bayesian linear discriminant analysis (LDA) (Yu et al., 2015), artificial neural networks (Yang et al., 2012), and genetic algorithms (Yang et al., 2012; Hasan et al., 2014; Kee et al., 2012). Recent work also demonstrates the efficacy of swarm-intelligence and hybrid meta-heuristics (Bhandari et al., 2024)—including particle swarm optimisation (PSO), binary PSO (Arican and Polat, 2020), multi-objective PSO (Chaurasiya et al., 2018), and hybrid meta-heuristic optimisation (HMO) (Mart́ınez-Cagigal et al., 2018).

Although numerous electrode selection methods have been proposed, relatively few have been evaluated for individuals with brain abnormalities (Bhandari et al., 2024). To address this gap, the current study investigates whether individualizing electrode locations can enhance P300-based BCI performance by better aligning with the atypical neuroanatomical and neurophysiological profiles commonly observed in participants with cerebral palsy (CP). Specifically, this study is an offline investigation of the effect of a custom individualized electrode subset on P300-BCI accuracy for participants with severe CP, mild CP, and typically developing controls. We hypothesize that people with CP can benefit from electrode selection by 1) improvement in BCI accuracy; and 2) the ability to use a smaller electrode subset size without jeopardizing performance. To our knowledge there is no systematic analysis of P300-design BCI accuracy using electrode selection for populations with CP in the existing literature.

## 2 Methods

### 2.1 Data for analysis

We conducted offline analysis on data from two different protocols of BCI use to access a 4-choice vocabulary test. Data in Protocol 1 was collected from participants with mild CP and typically developing controls (Warschausky et al., 2022). Protocol 2 involved participants with severe CP (Huggins et al., 2022). In particular, participants in Protocol 1 were required to have sufficient physical abilities to take the standard, paper version of a forced-choice vocabulary test. This meant that they were required to either be able to say the number of the desired answer or to reliably point to the answer. Participants were eligible for Protocol 2 only if they were unable to independently complete the paper-based version of the multiple-choice test and therefore could potentially benefit from a BCI-based alternative.

**Figure 1:**
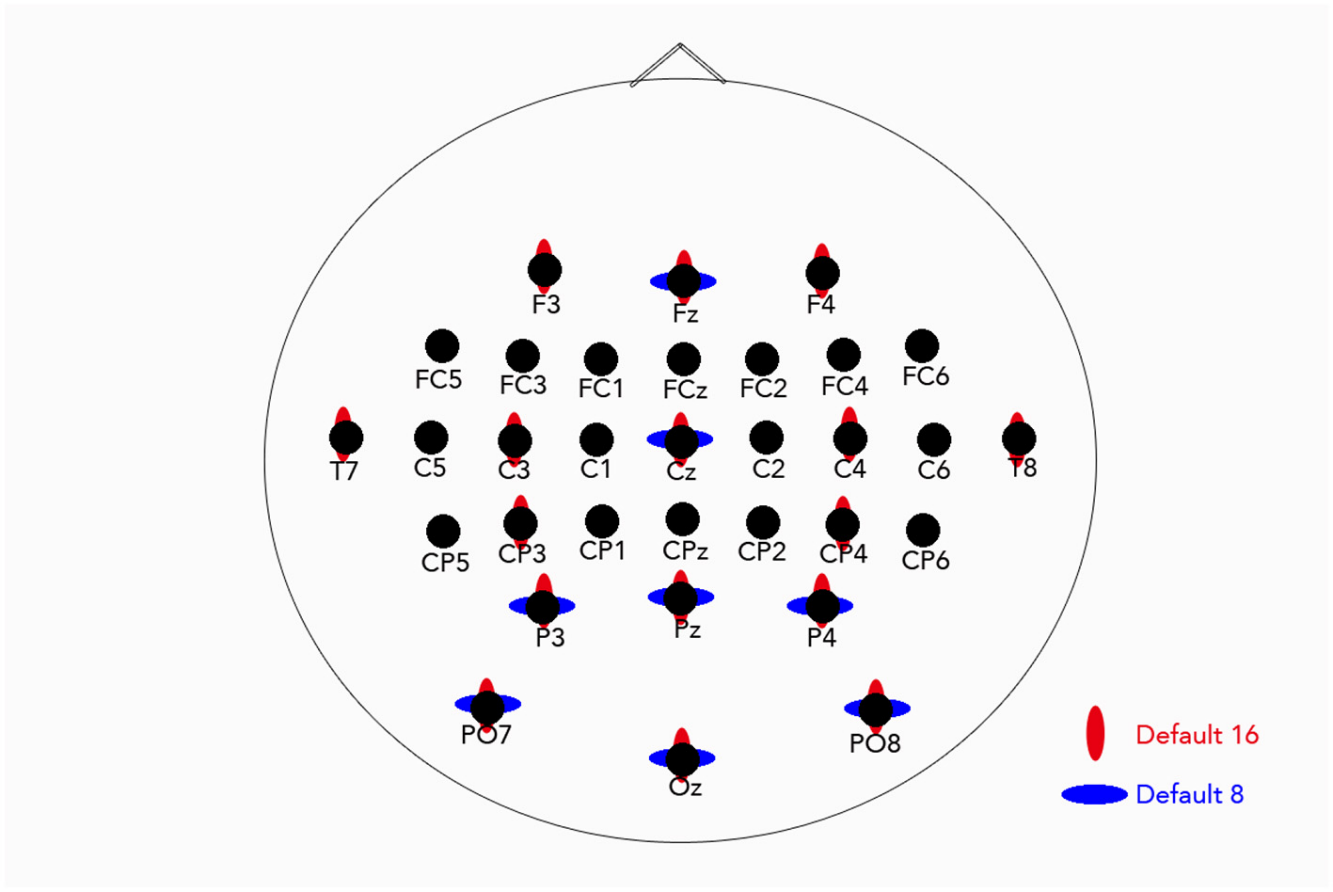
Locations of the 32 electrodes with the default 16 (Huggins et al., 2016; Thompson et al., 2014, 2012; McCann et al., 2015) and default 8 (Wolpaw et al., 2018) electrodes marked.

**Figure 2:**
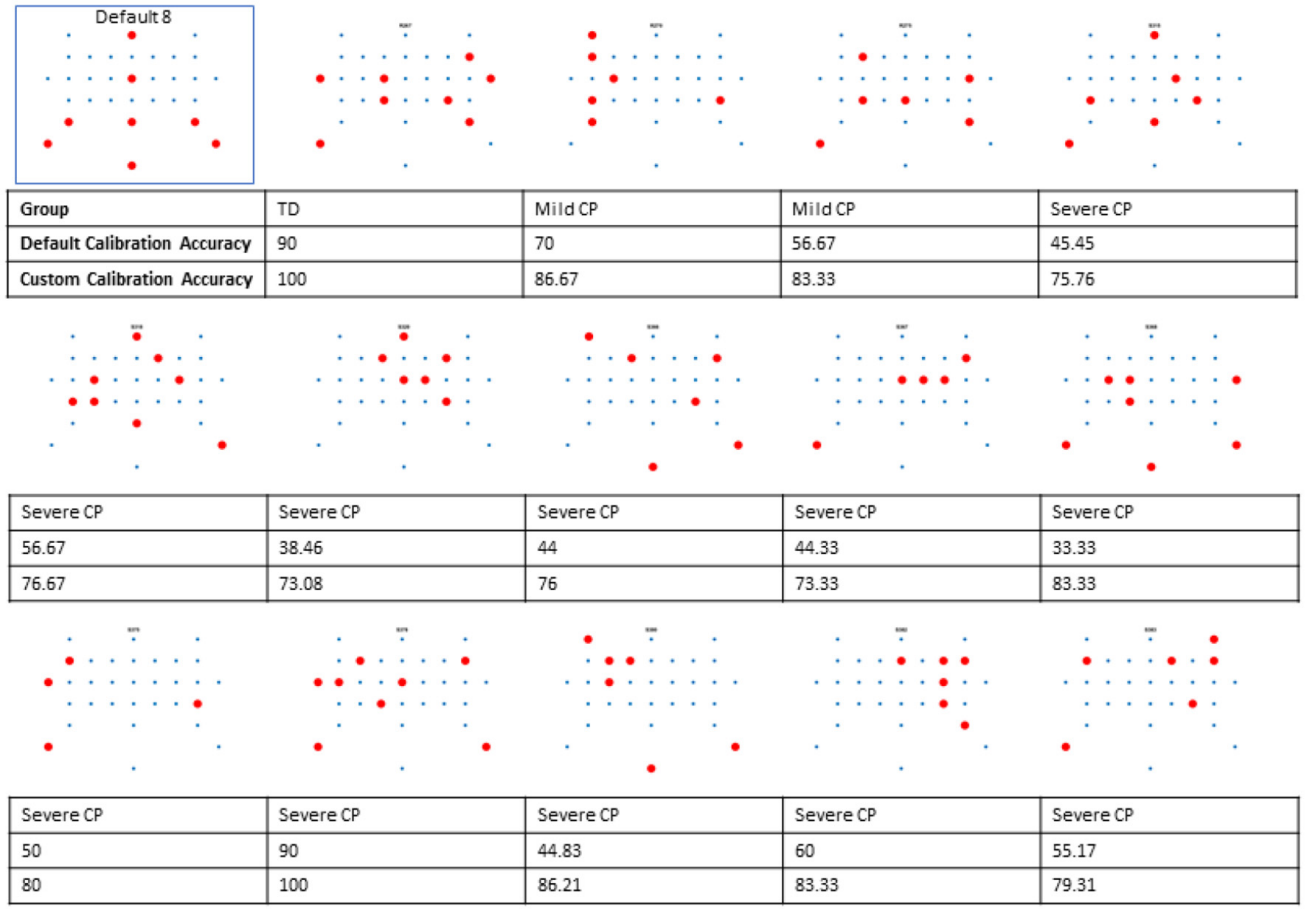
Custom subset locations, default and calibration accuracy for each participant with significant improvement. Together they show the degree of variability of custom electrode locations and the resulting changes in BCI performance.

In both protocols, data was collected from an Electro-Cap International, Inc., 32 location wet electrode (shown in Figure 1) EEG cap (F3, Fz, F4, FC5, FC3, FC1, FCz, FC2, FC4, FC6, T7, C5, C3, C1, Cz, C2, C4, C6, T8, CP5, CP3, CP1, CPz, CP2, CP4, CP6, P3, Pz, P4, PO7, PO8, Oz), with only 16 electrodes (default 16 in Figure 2) used for in-session calibration (F3, Fz, F4, T7, C3, Cz, C4, T8, CP3, CP4, P3, Pz, P4, PO7, PO8, Oz). Calibration was done with the step-wise linear discriminant analysis (SWLDA) classification from the BCI2000 (version 3) P300 Classifier program. EEG was recorded with two synchronized g.USBamp EEG amplifiers (Guger Technologies, Austria).

Each question (trial) of the vocabulary testing format included the audio presentation of a recorded word (audio prompt) and the presentation on-screen of four pictures. Literacy was not an inclusion/exclusion criteria and no words were presented in written form. Words for calibration were selected to be easy to match to the pictures. During recording of the calibration data, the user's attention was drawn to the picture that matched the audible word by leaving that picture in color and changing the other pictures to grayscale. Each picture had a labeled corner in which gray text on a black background intensified (flashed) to bright white as the stimulus to elicit the P300. The labels flashed individually in a pseudo-random order. Each label flashed once before any label repeated. The EEG after each flash was analyzed for the presence or absence of a P300 response (see Appendix Figure A1). Each label was flashed 10 times for each trial.

#### 2.1.1 Differences between protocols

**Figure 3:**
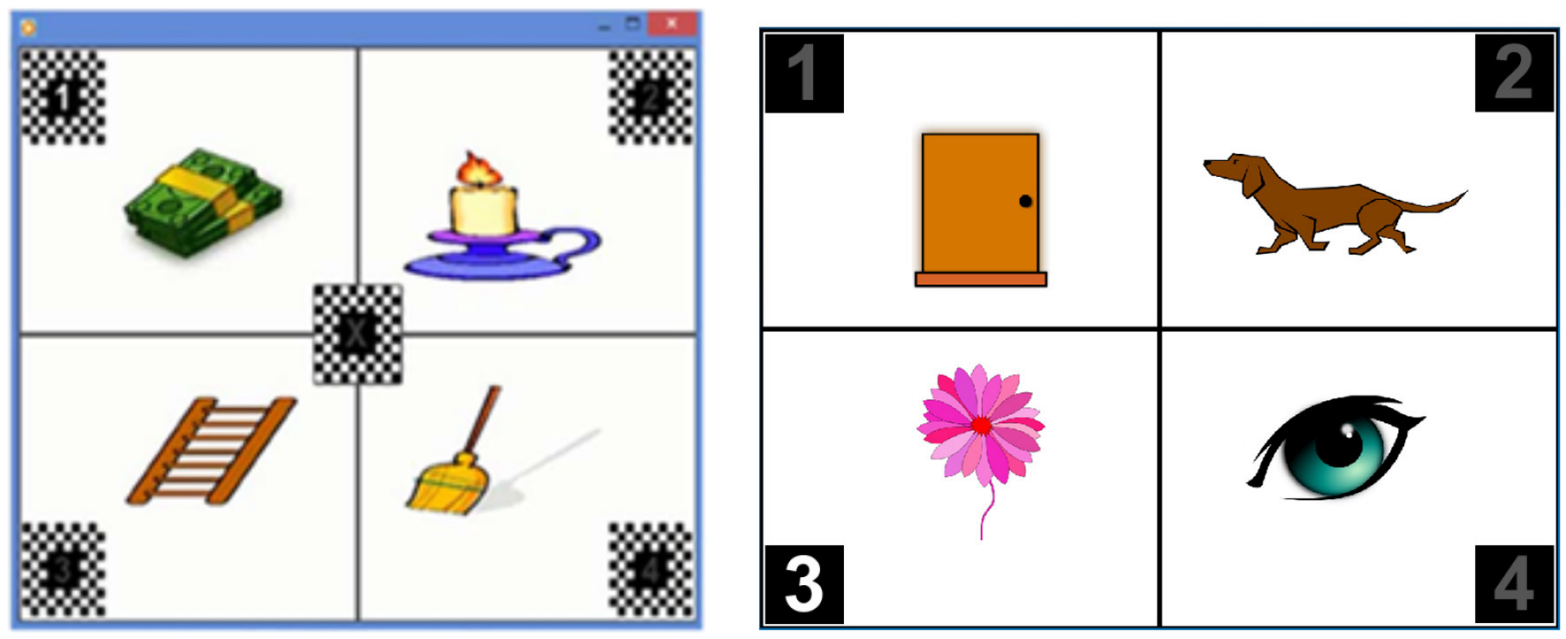
Screen layout for the BCI-presented vocabulary test format for **Left**: Protocol 1; **Right**: Protocol 2.

The P300 stimuli for Protocol 1 had an additional continuously flickering checkerboard border (Figure 3, **left**) that was intended to elicit a steady-state visual evoked potential (SSVEP). Offline SSVEP analysis of the Protocol 1 data did not show significant SSVEP responses and incorrect monitor refresh rates during data collection were suspected. This border was not used in Protocol 2 (Figure 3, **right**). Stimulus durations were different as a result of the different sample rates but were matched as closely as possible (duty cycle of 166.67ms for Protocol 1 vs. 156.25ms for Protocol 2). P300 stimulus flashes for Protocol 1 were 50ms in length with 116.7ms between flashes. P300 stimulus flashes for Protocol 2 were 62.5ms in length with 93.75ms between flashes. Inclusion criteria for Protocol 1 were ages 8-29 years. The original recruitment age for Protocol 2 was originally restricted to 10-29 years, but the upper bound was later relaxed to 45 years.

Audio prompts for Protocol 1 had only the word to find. Audio prompts for Protocol 2 included the instruction “Find” as well as the word. The words and images used for the protocols were different since Protocol 2 words were carefully selected for awareness by those with severe impairments.

In Protocol 1, EEG was sampled at 600 Hz and calibration data consisted of at least 60 trials in separate files of 30 trials each. In Protocol 1, quality of the EEG responses was checked by attempting SWLDA calibration after completion of the first calibration file (30 trials). Some participants were unable to complete a calibration run due to insufficient task engagement during the calibration procedure. If the participant was not paying attention or the highest reported accuracy was < 75%, the calibration task was discussed with the participant to clarify the instructions and then the first calibration file was repeated. Further attempts to obtain two usable calibration data files were dependent on the participant's willingness and the experimenter's perception of the participant's attention to the task. Participants who were unwilling to continue or who did not attend to the calibration task exited the study without completing calibration. Participants who did not complete calibration attempted between 1 and 4 calibration files (30 and 120 trials).

In Protocol 2, EEG was sampled at 256 Hz and calibration data was intended to include 30 trials. These trials were separated into shorter trial blocks intended to accommodate limited attention spans. By default, these were presented in 3 files of 13 trials, 13 trials and 4 trials. However, they were also available in files of 4 trials each to accommodate those with limited attention span. The experimenter could select the number of trials per file to accommodate the participants' attention span with a goal of getting 30 calibration trials. Participants encountered challenges to BCI use and calibration, as described in Huggins et al. (2022). Those who attempted calibration did between 13 and 94 trials of calibration data. As in Protocol 1, continued participation depended on the participant's willingness and the experimenter's perception of their attention to the task.

### 2.2 Participants in Protocol 1

Data were obtained from 17 participants with CP (5 females and 12 males, age 14.7±5.6 years) and 10 typically developing participants (6 females and 4 males, age 14.7±4.3 years) who were part of a study on the use of a BCI-adapted multiple choice test, described in Alcaide-Aguirre et al. (2017). The Institutional Review Board of the University of Michigan approved the protocol (HUM00012968). Inclusion criteria for both groups were sufficient vision and sufficient speech or movement to participate in the standardized version of the Peabody Picture Vocabulary Test Fourth Edition (PPVT-IV) (Dunn and Dunn, 2007). Exclusion criteria included (1) history of moderate or severe acquired brain injury or other major neurological condition such as stroke, encephalitis, or refractory seizure disorder (for those with CP, this refers to events subsequent to the onset and diagnosis of CP), (2) major psychiatric disorder such as major depression, severe anxiety or psychosis that precluded participation, and (3) for those under age 18, inability of the parent/guardian to complete a child history. In the group with CP, one participant was taking baclofen and one was taking sertraline. In the typically developing group, one participant was taking sertraline.

In the group with CP, primary tone in all participants was spasticity, 60.0% had hemiplegia and 40% diplegia. Functional classifications were obtained with the Gross Motor Functional Classification System (GMFCS) (Palisano et al., 2008), the Manual Ability Classification System (MACS) (Eliasson et al., 2006), and the Communication Function Classification System (CFCS) (Hidecker et al., 2011). Score distributions are described in Table 1.

**Table 1:** Distributions of participants by functional classification sytem in Protocol 1 and Protocol 2.

		**Level I**	**Level II**	**Level III**	**Level IV**	**Level V**	**Did not report**
Protocol 1 (Mild CP)	GMFCS	64.7%	-	11.8%	5.9%	5.9%	11.8%
	MACS	23.5%	47.1%	11.8%	11.8%	-	5.9%
	CFCS	70.6%	11.8%	5.9%	5.9%	-	5.9%
Protocol 2 (Severe CP)	GMFCS	-	-	-	16.7%	83.3%	-
	MACS	-	-	-	8.3%	91.7%	-
	CFCS	-	4.2%	-	45.8%	50%	-

For participants who reported more than one level for a classification system, they are categorized into the less severe level.

Among the participants, 10 individuals with CP (2 females and 8 males; age 17.4 ± 5.5 years) and all typically developing controls completed the study, while seven individuals with CP (3 females and 4 males; age 10.9 ± 3.0 years) were unable to calibrate the BCI despite repeated calibration attempts. This subgroup was characterized by a lower mean age compared to participants who completed the study. In addition, one participant with CP who was able to use the BCI only had 30 trials of calibration data. All participants in Protocol 1 are included in the electrode selection calibration analysis as detailed in Section 2.6; only participants who had complete data (nine participants with CP, 10 participants not affected by CP) are included in the testing analysis as detailed in Section 2.7.

### 2.3 Participants in protocol 2

In Protocol 2, usable BCI calibration data from 24 participants with severe CP (16 females and 8 males, age 18.3±8.4 years) were obtained. Participants were part of a study comparing a BCI-access to an eye-gaze-interface-access multiple choice test. The Institutional Review Board of the University of Michigan approved the protocol (HUM00130187) as did the Cerebral Palsy Alliance Human Research Ethics Committee (approval number: 2017_05_02). The participants for this study were required to be unable to access the paper version of the multiple choice test.

Sixteen participants were from the USA and 8 from Australia. Primary tone for 17 participants (70.8%) was spasticity, four participants (16.7%) was dystonia, and three participants (12.5%) had diagnoses of both spasticity and dystonia. Participant characteristics are described in Table 1.

Among the 24 participants, there were five for whom calibration with the 16 electrodes used for the online study met the performance threshold of 75% accuracy for successful calibration. Due to the limited number of calibration trials collected in Protocol 2 and the absence of sufficient testing data, participants enrolled under this protocol could not be included in the testing analyses. Challenges related to attention, fatigue, and sustained engagement during calibration, previously described in Huggins et al. (2022), limited the feasibility of obtaining data suitable for downstream evaluation.

### 2.4 Offline data preprocessing

All data were processed using a modified version of the BCI2000 (v3) P300Classifier tool (Schalk et al., 2004). The original BCI2000 P300Classifier pipeline and its SWLDA implementation were preserved, with two additions: (i) an option to train and evaluate the classifier using a custom electrode subset of a specified size, and (ii) linear detrending applied within each ERP response window in preprocessing prior to feature extraction.

The BCI2000 P300 Classifier calibration pipeline for a specific set of electrodes proceeded as follows. First, the continuous EEG for the specified electrodes was segmented into stimulus-locked epochs and labeled as target or non-target based on whether the flash corresponded to the target picture. Second, features were extracted from each epoch within an ERP window of 0-800 ms post-stimulus. The resulting feature vectors were decimated to 20 Hz (down-sampled) to reduce dimensionality and improve computational efficiency. Third, a SWLDA model was trained to discriminate target from non-target epochs. During SWLDA, features are added to the model if their statistical association with the class label meets the pEnter criterion and are removed if they no longer meet the pRemove criterion. For all analyses, SWLDA was configured with a maximum of 60 features, with pEnter = 0.10 and pRemove = 0.15, consistent with common P300 speller parameterizations (Colwell et al., 2014).

In the Protocol 2 data, the amount of calibration data varied across participants due to difficulties in the online session with maintaining participant attention or in calibration. Some participants have fewer than 30 trials recorded, while some others have extra calibration data in the attempt to calibrate the BCI. Upon inspection, we found that the data was also more noisy than Protocol 1 data. We therefore applied 2 additional steps for preprocessing: (1) we chose a calibration file combination so that the total number of trials is as close to 30 as possible, and (2) we first tried to calibrate without spatial filtering, and if neither the default nor custom subset could calibrate, we applied a common average reference (CAR) filter.

### 2.5 Electrode selection

A custom subset of eight electrodes was selected from the 32 available channels. The choice of eight electrodes was motivated by both practical and empirical considerations. We adopted the same default eight-electrode configuration used by Wolpaw et al. (2018) and Holz et al. (2015), who demonstrated that this setup is appropriate and realistic for home-use P300 BCI applications. Accordingly, an eight-electrode subset represents a reasonable trade-off between system performance and setup time. In addition, at the time of this study, commercially available EEG amplifiers commonly supported eight channels, making this configuration a natural and practically relevant choice.

For the electrode subset selection, we implemented the forward selection method proposed by McCann et al. (2015), which was shown to be as effective as exhaustive search when generalized to testing data. The forward selection algorithm added electrodes one-by-one that would locally maximize accuracy until the desired subset size was reached. Initially, all available electrodes were in the “available” set and the “chosen” set was empty. Electrodes were moved to the “chosen” set one at a time, based on the calibration accuracies for the addition of one more electrode. Thus, each iteration obtained the calibration accuracies of all candidate subsets, each composed of all electrodes in the current “chosen” set plus one electrode in the “available” set. The candidate subset with the highest accuracy would be the new “chosen” set, and the corresponding electrode from the “available” set would be excluded from the new “available” set.

### 2.6 Calibration

Calibration was done separately on data from each participant. For consistency in data size across the two protocols, thirty trials from Protocol 1 were used. Calibration accuracy for the custom electrode subset and the default subset was compared. In case of a tie in accuracy between the custom and default subset when all 10 flashes were used, we decreased the number of flashes from 10 and reported the accuracy at the first number of flashes when the tie was broken.

### 2.7 Generalization testing

The larger amounts of calibration data (N = 60 trials) available for Protocol 1 enabled additional investigation of the ability of an electrode subset derived from calibration data to generalize to new data. For this investigation, 20 trials were selected randomly and held out for testing while the remaining 40 trials were used for calibration. Electrode selection using the 40 calibration trials was done with an iterative process. The electrode subset was treated as a hyper-parameter and fit via a 40-fold procedure: in each fold, one trial was excluded from the 40-trial calibration set. Let EEG electrode cap size = S (32 electrodes), calibration data = N (40 trials) with a target custom electrode subset size = M. For each participant and each fold, one trial was held out, and the remaining 39 (N-1) trials were fitted with SWLDA with the forward algorithm for M electrodes. A total of 40 (N) electrode subsets through the 40 (N) folds were obtained with the process. Then, each electrode received a score based on the order (k) in which it was added to the subset. Specifically, we found the top M highest scoring electrodes calculated by $\overset{N}{\underset{n=0}{\sum }}\overset{S}{\underset{s=0}{\sum }}max\left(0,M-k_{n,s}+1\right)$ as the custom electrode subset for the participant. This produced an electrode subset of size 8 (M), which was then used to obtain weights on the entire 40 (N) trials of the calibration data. The weights were then applied to the held-out 20 trials to determine testing accuracy. Both calibration and testing accuracy were reported for participants in Protocol 1 who had complete data (9 participants with mild CP, 10 typically developing).

### 2.8 Statistical analysis

Statistical analysis to assess the group effects and individual effects of the custom electrode subset was done in R version 4.0.4 (R Core Team, 2021).

#### 2.8.1 Group effects

A generalized linear mixed-effects model (GLMM) with poisson outcome was used to evaluate age-controlled group effects of electrode selection in both the calibration data and the testing data, with subset type (default or custom), group (typically developing controls, mild CP, severe CP), and age as fixed effects, and individual participant as a random effect.

A linear mixed-effects model was used to assess whether the effect of electrode subset seen in calibration accuracy generalizes to accuracy on testing data. This analysis was done for the participants with complete data (10 people with mild CP, 10 typically developing controls). Difference in accuracy at 10 flashes (custom - default) was evaluated against the default subset accuracy, age, and the interaction between group and analysis paradigm (calibration vs testing).

#### 2.8.2 Individual effects

We assessed the statistical significance of custom electrode subset at the individual level. For each participant, calibration data of an average of 30 trials from the default 8 electrodes was fitted to a binomial distribution using the maximum likelihood estimation (MLE) method. It was assumed that each trial was independent and identically distributed (i.d.d.). Let the number of random trials = n, and the number of correct classifications using the default subset = k. We calculated the cumulative probability *P*(*X*≥*k*) from the custom 8 electrodes with respect to the fitted binomial distribution. This statistic translates as “using the custom subset, in n random trials, the probability of seeing greater or equal to k success”, which entails whether a custom subset improves BCI accuracy when compared to the default subset. The cumulative probability is equivalent to a one-sided p-value using the binomial distribution.

### 2.9 ERP visualization

To provide qualitative context for the electrode selection results, event-related potentials (ERPs) were visualized for representative participants from each group. For each participant, stimulus-locked EEG epochs were averaged separately for target and non-target stimuli at each electrode over a 0-1000 ms post-stimulus window. Prior to averaging, each epoch was linearly detrended and mean-centered. These ERP visualizations were included for illustrative purposes only and were not used as quantitative outcome measures.

## 3 Results

### 3.1 Calibration accuracy

**Figure 4:**
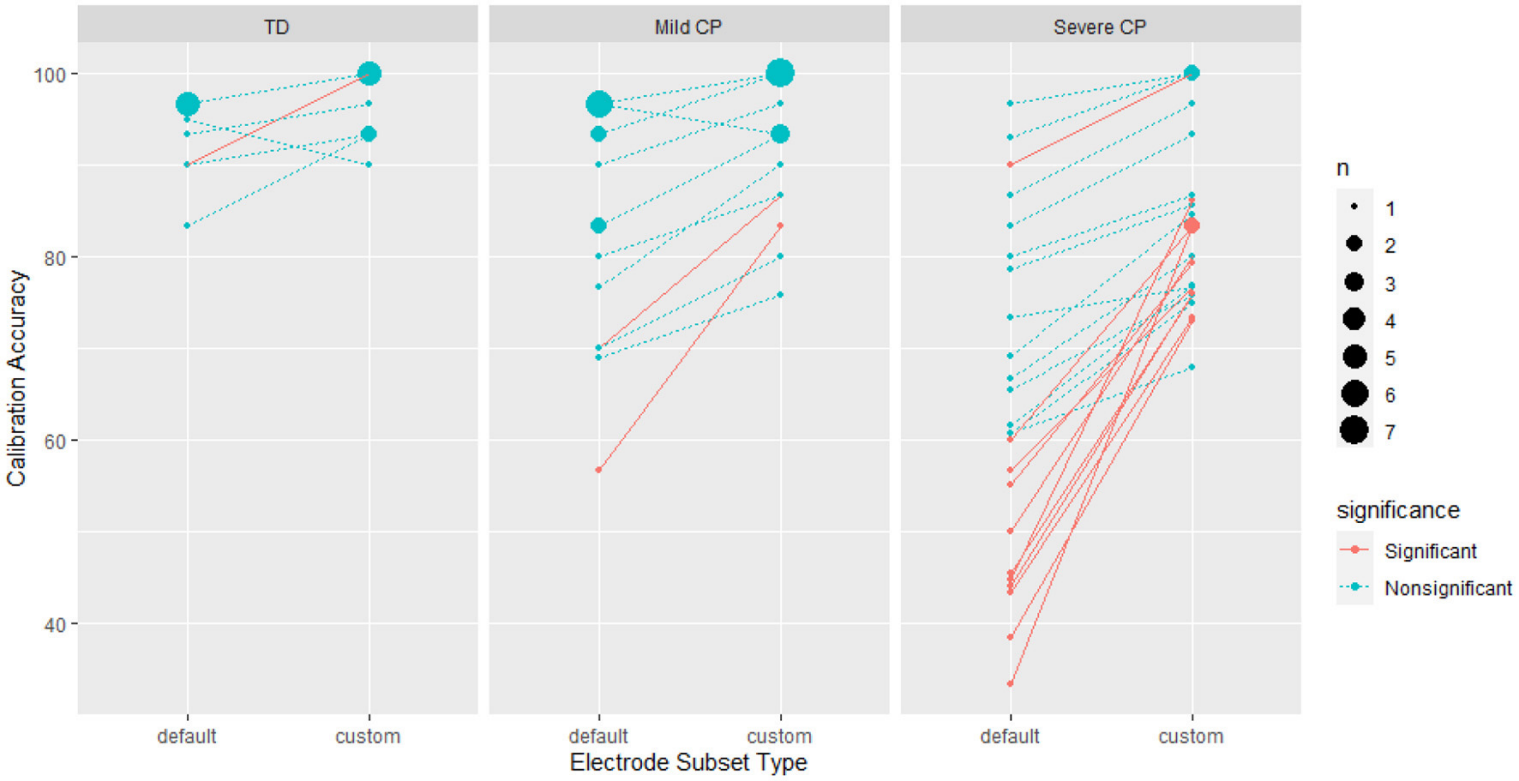
BCI calibration accuracy with different electrode subset types by participant group. The solid pink lines represent individuals with a significant improvement calculated as described in Section 2.8.2. The size of the data points represents the number of participants with the same accuracy score. Note, that the magnitude of improvement is not a complete representation of the statistical significance.

Between group comparison showed that custom electrode subset significantly improved BCI calibration accuracy for the severe CP group (p < 0.0001), but not for the mild CP group (*p* = 0.185) nor the controls (*p* = 0.676). For the severe CP group, the average accuracy using the custom subset improved by 28.6% (95% CI [13.4%, 46.1%]) compared to the default subset. Using the default subset, there was no significant difference in calibration accuracy between the mild CP group and the controls (p = 0.208), but the BCI accuracy was significantly worse in the severe CP group than the controls (*p* < 0.0001). Using the custom subset, there was no significant difference between the mild CP and the severe CP group (p = 0.522), but the BCI for the control group performed significantly better (*p* = 0.0005). Figure 4 shows changes in calibration accuracy by electrode subset (custom vs default) for individuals in each group.

**Figure 5:**
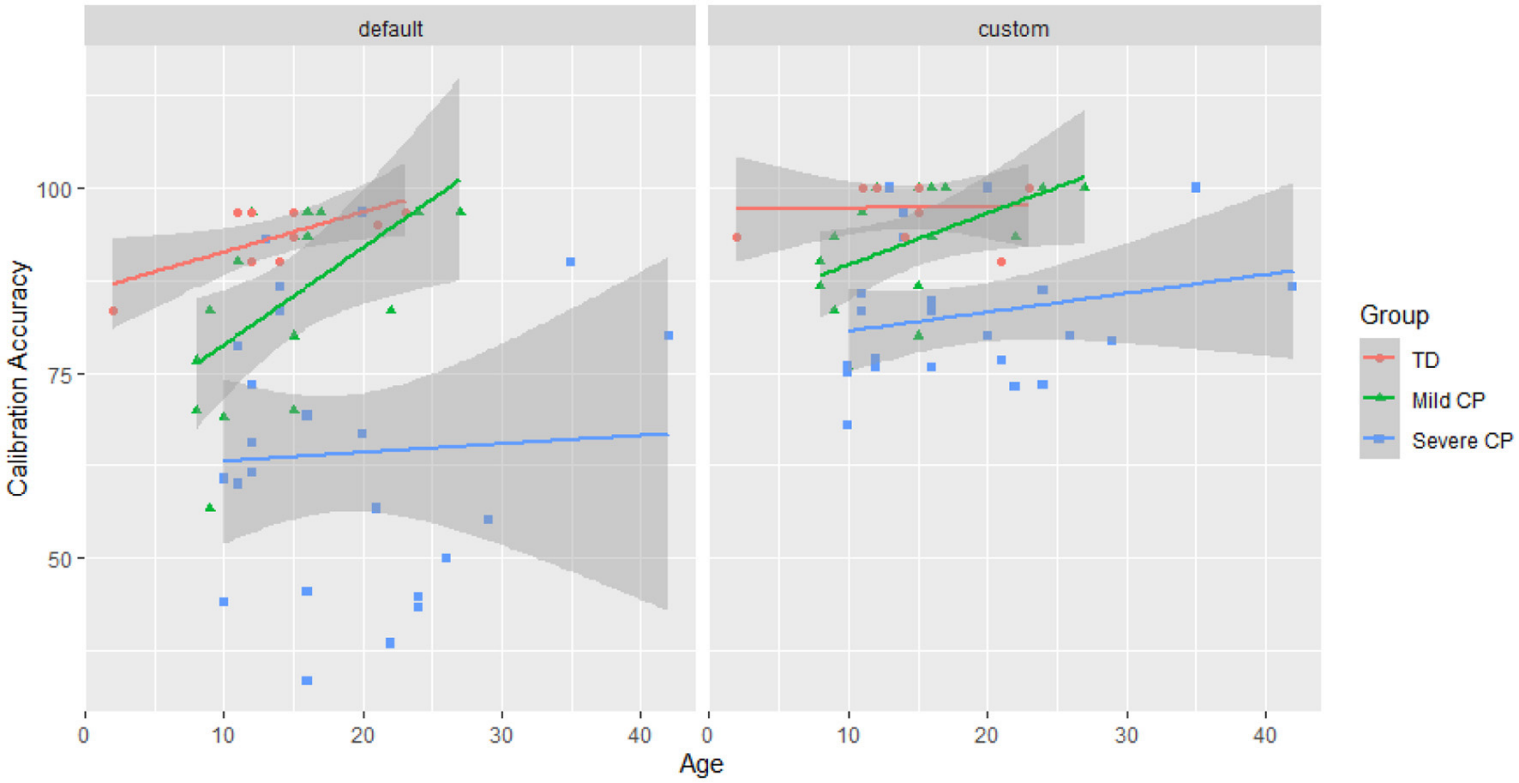
BCI calibration accuracy across age. Experimental data is plotted as scatters and a fitted line is plotted for each group for each subset type. The shaded area represents the 95% confidence interval.

At the group level, within group comparison shows that age significantly affected calibration accuracy (*p* = 0.020) (Figure 5).

For individual effects, electrode selection produced significant improvement in calibration accuracy for one control (out of 10 participants), two people with mild CP (out of 17 participants), and 11 people with severe CP (out of 24 participants) (see Figures 2, 4). It is worth noting that the two people with mild CP who had significant improvement did not meet minimum calibration accuracies with the default subset during the data collection to proceed to testing.

Nine of 17 participants (52.9%) with spasticity and mixed spasticity-dystonia (66.7%)participants with mixed spasticity-dystonia showed significant improvement in 2 of 3 participants (66.7 %) with customized electrode selection, whereas no participants with dystonia alone demonstrated significant improvement.

### 3.2 Generalization to testing data

Neither the calibration accuracy with the default subset (*p* = 0.160) nor age (*p* = 0.488) significantly predicted the change in BCI accuracy from calibration to testing by different subsets (default vs custom).

For the controls, there was a significant reduction (−9.58%, 95% CI [−13.33%, −5.84%]) in the effect of the subset during testing compared to calibration (*p* < 0.0001). There was no evidence that the effect of electrode subset on testing accuracy was different from its effect on calibration accuracy in the mild CP group (*p* = 0.556).

**Figure 6:**
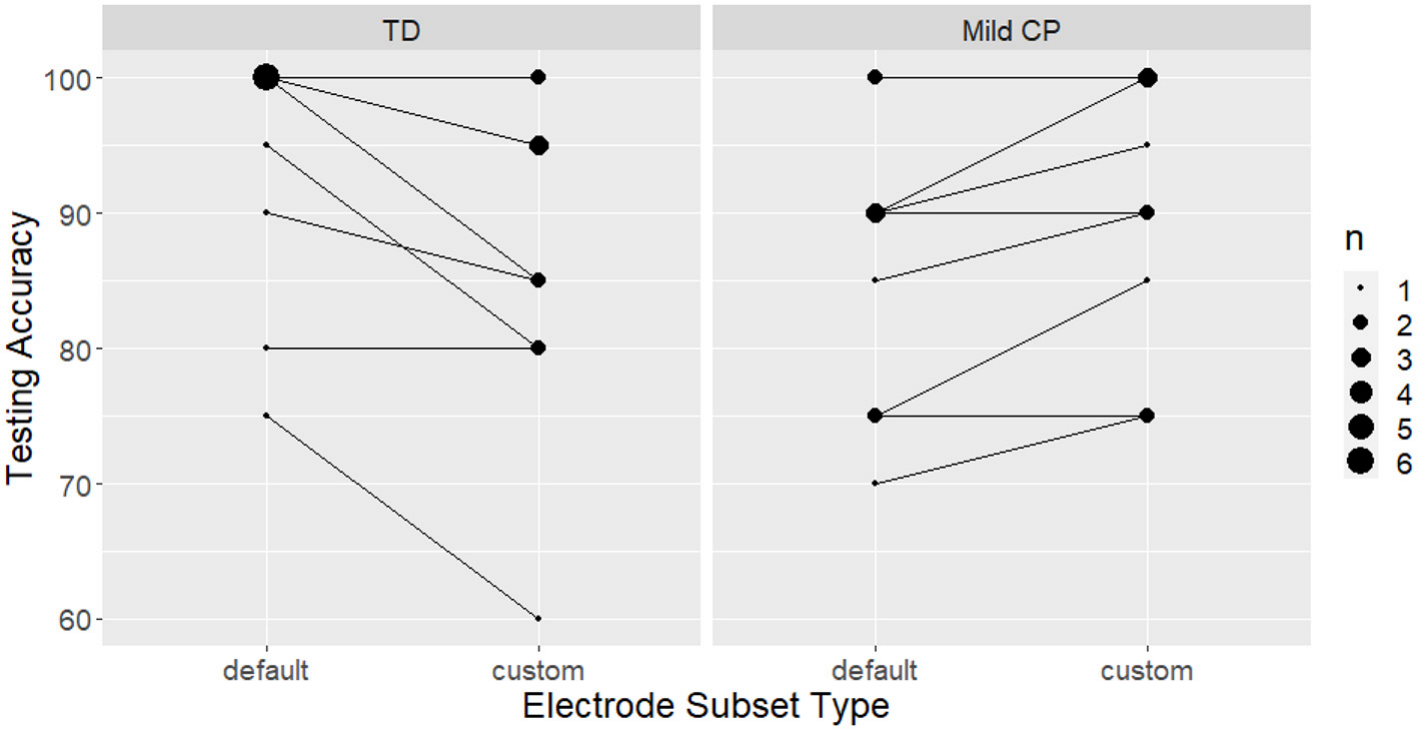
BCI testing accuracy with different electrode subset types. No significant improvement is found at the individual nor the group level. The size of the data points represents the number of participants with the same accuracy score.

We evaluated significance of group effects of electrode selection on testing accuracy. Comparisons were done using the number of flashes where ties are broken. Neither the mild CP (*p* = 0.694) nor the control group (*p* = 0.495) showed significant difference in testing accuracy between using the custom and default subset (Figure 6). We could not evaluate the custom subsets for the severe CP group because testing data was not available.

### 3.3 Number of useful electrodes

**Figure 7:**
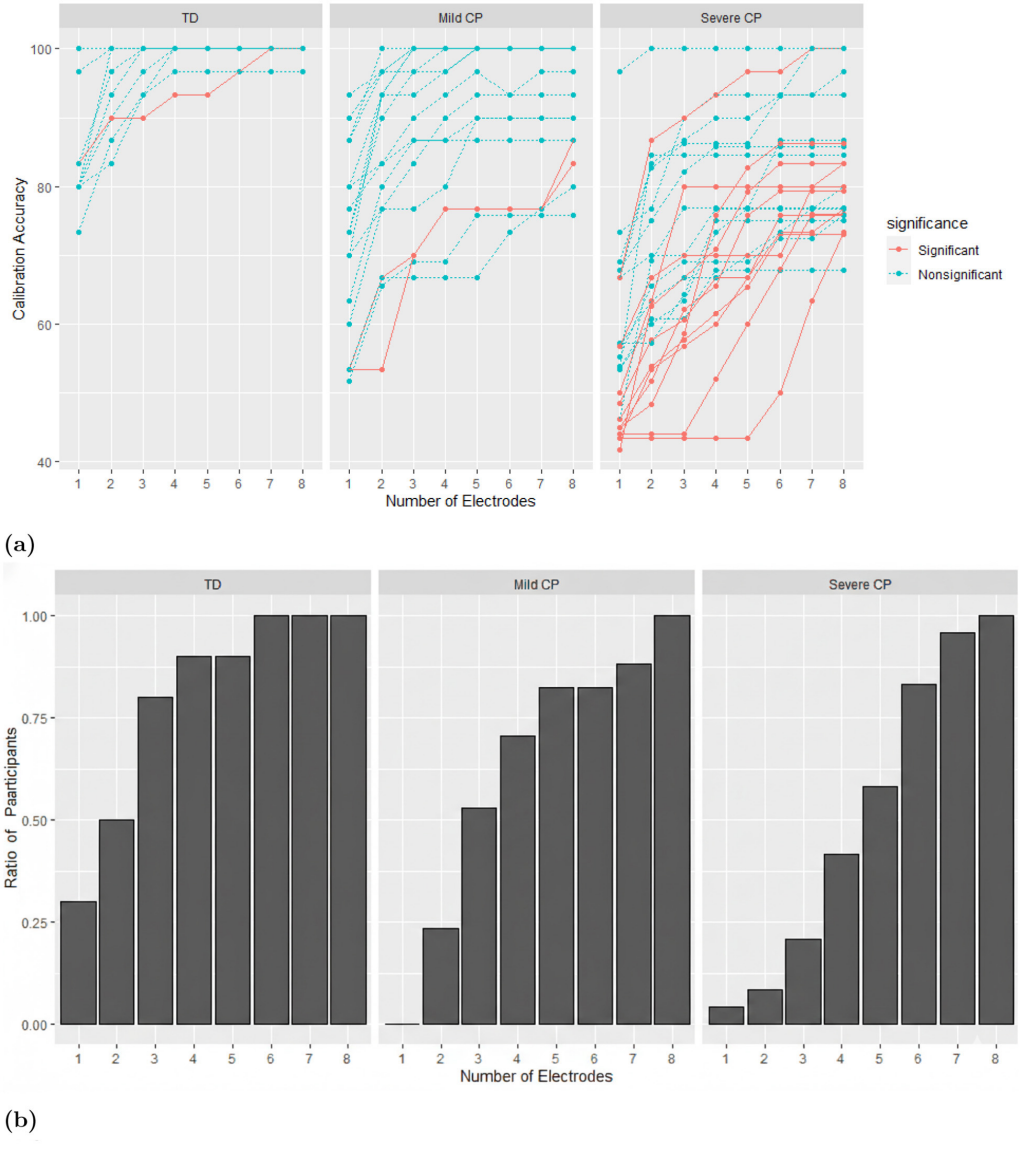
Calibration accuracy for custom electrode subset sizes. **(a)** Each line is a participant. Accuracies are plotted from 0 to 8 selected custom electrodes. **(b)** Percentage of participants in each group who reach at least 95% of their own calibration accuracy at 8 electrodes using the number of electrodes specified in the x-axis.

Differences in calibration accuracy as the size of custom electrode subset increased were investigated up to 8 electrodes (Figure 7a). As found by McCann et al. (2015), most participants had an asymptotic increase in accuracy as electrode subset sizes increase. People with severe CP needed a bigger electrode subset size to achieve at least 95% of their own calibration accuracy at 8 electrodes (Figure 7b).

Overall, limiting the maximum electrode subset size to 8 is efficient. Of the 14 people who saw improved performance for the custom electrodes, 11 of them did not use all 8 custom electrodes. While the remaining 3 might have gotten some benefit from additional electrodes, for most people a maximum of 8 electrodes is sufficient.

### 3.4 ERP characteristics at selected electrodes

Appendix Figure A1 shows average ERPs for representative participants from each group. Selected electrodes are highlighted in red. In these channels, target and non-target responses exhibit clearer visual separation relative to non-selected electrodes, with more pronounced deflections from baseline following target stimuli.

Across participants, ERP waveforms from the severe CP group appeared noisier than those from the mild CP and control groups. In addition, the spatial distribution of discernible ERP responses in the severe CP participant was more restricted, whereas participants in the control and mild CP groups showed broader spatial distributions of similar ERP morphology.

## 4 Discussion

The findings indicate that an individualized electrode subset can be essential for BCI calibration for people with severe CP, and an electrode subset can benefit people with mild CP. However, the default electrode subset is adequate or even preferred for typically developed individuals. Figure 5 shows that the improvements from the custom electrode subset are not limited to a particular age.

The etiology and pathophysiology of CP may explain the benefit gained from a custom electrode subset. CP stems from early brain lesions and can result in neurophysiological differences including differences in representation of functions (Rai et al., 2013; Qin et al., 2018). For example, people with spastic CP often show evidence of periventricular leukomalacia (PVL) (Hoon, 2005). PVL has been associated with altered connectivity including reduced posterior pathways to parietal and occipital regions. Therefore, electrode selection may identify better custom locations to record informative EEG to accommodate those differences. Our result suggests that there are electrodes that are not in the default subset that are in fact informative for some participants with CP, and our electrode selection algorithm is able to identify those electrodes.

### 4.1 Selected electrodes and ERPs

Figure 2 illustrates the individualized electrode subsets for participants whose BCI calibration accuracy improved significantly with electrode selection. For some participants with CP, the selected electrodes were strongly lateralized, highlighting the degree of inter-individual variability in optimal electrode placement.

ERP visualizations (Appendix Figure A1) provide qualitative context for these findings. In participants with severe CP, ERP responses were more spatially restricted and exhibited greater noise, consistent with movement artifacts and variable attention observed during data collection. This restricted distribution suggests increased sensitivity to electrode placement, such that performance improvements depend more strongly on selecting a small subset of informative channels. In contrast, participants in the control and mild CP groups exhibited broader spatial distributions of similar ERP morphology, suggesting that electrode locations may be more interchangeable for these individuals.

### 4.2 Number of useful electrodes

McCann et al. (2015) found minimal improvements in BCI accuracy for electrode subsets larger than 4 electrodes. Our results with the typically developing group support that finding, since at subset size 4, 9 out of 10 control participants were within 95% of their maximum accuracy and all had reached that threshold by subset size 6. However, this conclusion cannot generalize to people with CP, as Figure 7b shows that over 50% of the participants with severe CP benefit from using more than 4 electrodes and for some a subset of 8 electrodes is more effective than 7.

### 4.3 Electrode selection for typically developing individuals

Colwell et al. (2014) reported significant performance improvements for typically developing individuals using sequential forward electrode selection. In contrast, we did not observe a comparable benefit of electrode selection for typically developing participants in the present study. This discrepancy likely reflects substantive methodological differences between the two studies, as well as differences in the study objectives. Whereas Colwell et al. examined whether a single optimized electrode set could improve BCI performance across participants, our study focused on assessing the necessity and benefit of individualized electrode subsets tailored to each participant.

Several key methodological differences may account for the differing results. First, the experimental paradigms differed substantially. Colwell et al. employed a P300 speller using a 9 × 8 checkerboard paradigm with 38 character selections during calibration, whereas our study used a four-question multiple-choice paradigm with individual stimulus flashes. Differences in task structure, stimulus timing, and cognitive demands may influence ERP characteristics and, consequently, the potential benefit of electrode selection.

Second, the electrode search spaces differed between studies. Our electrode montage was designed to provide greater coverage of motor-related cortical areas to accommodate individuals with spastic CP. In contrast, the electrode configuration used by Colwell et al. included a greater number of posterior electrodes, and many of the electrodes identified as most informative for typically developing participants in their study were located in posterior regions. In our study, all posterior electrodes were already included in the default electrode subset, limiting the opportunity for electrode selection to further improve performance for typically developing participants by adding additional posterior channels.

Finally, participant age may also have contributed to the observed differences. Although age was not reported in Colwell et al., the description of participants receiving course credit suggests a sample of college-aged young adults. By comparison, the typically developing participants in the present study were younger and spanned a wider age range (14.7 ± 4.3 years). Age-related differences in attention, cognitive processing, or ERP morphology may therefore have influenced the effectiveness of electrode selection.

Taken together, these differences suggest that the effectiveness of electrode selection may depend on the experimental task, the available electrode search space, and participant characteristics such as age. Accordingly, caution is warranted when generalizing electrode selection findings across different populations, experimental paradigms, and BCI systems.

Furthermore, a motor-imagery BCI study by Meng et al. (2018) reported that typically developing individuals did not benefit from electrode selection in online BCI use, despite observing improvements in offline analyses. The authors suggested that these offline gains may reflect non-task-related neural modulation captured by electrodes outside the targeted motor cortices, which could transiently improve performance within a single session but may not support stable skill acquisition or generalization to online control. Together with the present findings and those of Colwell et al. (2014), these mixed results indicate that there is no consistent physiological mechanism by which electrode selection reliably benefits typically developing individuals. In contrast to populations with atypical neuroanatomy, such as individuals with CP, the neural signals of typically developing users may be sufficiently robust and spatially distributed that electrode selection does not confer a consistent or generalizable advantage.

### 4.4 Limitations and future research directions

Several limitations of this study should be acknowledged, which also motivate important directions for future research. First, electrode selection was performed offline and was not available in real time during either experimental protocol. Indeed individualized electrode subsets to accommodate atypical neuroanatomy was hypothetical and preliminary data was needed to determine if additional study was warranted. As a result, participants did not directly benefit from individualized electrode subsets during data collection, and the reported performance improvements reflect retrospective analyses rather than online BCI use. These offline results indicate that future studies should evaluate customized electrode selection in online settings and across multiple sessions to assess its stability, usability, and impact on real-time BCI performance.

Second, EEG data collected from participants with severe CP were often affected by substantial noise arising from involuntary movements, muscle artifacts, and variable signal quality. Although additional preprocessing steps were applied to obtain usable data, such procedures may not always be feasible in real-world deployments. Future work should investigate whether individualized electrode selection can reduce sensitivity to noise or preprocessing demands, particularly in home or clinical environments.

Third, neuroimaging data were not available for participants in this study. Consequently, we could not directly relate selected electrodes or observed ERP components to individual neuroanatomy. Given the known heterogeneity in brain structure and function among individuals with CP, future studies combining EEG-based electrode selection with structural or functional neuroimaging could provide valuable insight into how individualized electrode configurations relate to underlying neuroanatomy and cortical organization.

Finally, the forward greedy search algorithm used for electrode selection is not considered state-of-the-art relative to more recent optimization or learning-based approaches. This method was intentionally chosen for its simplicity, computational efficiency, and previously demonstrated performance comparable to exhaustive search in P300 BCI applications (McCann et al., 2015). In the offline analyses conducted in this study (Microsoft Windows 11 Enterprise; Intel i7 processor; 32 GB RAM), selecting an 8-electrode subset for a single participant required approximately 3 minutes using forward selection, compared to over 80 minutes for backward selection on the same dataset, highlighting its practical computational advantages. Importantly, the primary aim of this work was not to develop or evaluate a novel selection algorithm, but to assess the feasibility and impact of customized electrode placement for individuals with CP. Future research may benefit from incorporating more advanced selection methods and evaluating their performance in online BCI settings.

More broadly, individualized electrode selection should be viewed as one component of a multi-faceted approach to improving BCI accessibility for individuals with CP. Prior work has highlighted the significant attention, behavioral, and usability challenges faced by this population (Huggins et al., 2022). Future studies should investigate how personalized electrode configurations interact with other system-level adaptations, such as task design, feedback, and user-state monitoring, to support sustained BCI use (Huggins et al., 2015).

### 4.5 Future directions for clinical implementation

Several feasible deployment pathways can be envisioned for clinical implementation of individualized electrode selection. One approach is a tiered calibration strategy, in which users first attempt calibration using a standard, low-channel-count electrode configuration that is sufficient for a substantial proportion of users. If calibration is unsuccessful or performance is inadequate, a secondary session can be conducted using a higher-density electrode montage in a controlled laboratory setting to identify a customized electrode subset. Once identified, this personalized subset can be implemented using a reduced-channel, lower-cost EEG system for subsequent clinical or home use, minimizing setup time and cost for most users while retaining the option for customization when needed.

Alternatively, an initial clinical evaluation could begin with a higher-density electrode montage. In this case, a customized electrode subset would be identified offline using automated selection algorithms and then translated into a personalized low-channel configuration. Although this approach involves greater upfront cost and setup time, it may be appropriate in specialized clinical or research settings where individualized optimization is a priority. Future studies should examine whether neuroimaging can predict the efficacy of customized electrode subsets.

## 5 Conclusion

The study shows for the first time that people with severe CP can benefit significantly from electrode subset selection.

## Data Availability

The raw data supporting the conclusions of this article will be made available by the authors, without undue reservation.
