# Supplementary material for: Individualized electrode subset improves the calibration accuracy of an EEG P300-design brain-computer interface for people with severe cerebral palsy
Source: Front Hum Neurosci. 2026 Mar 26;20:1720969. doi: 10.3389/fnhum.2026.1720969 (PMC13061863; doi:10.3389/fnhum.2026.1720969)
Supplement: Supplementary file 1 [file Data_Sheet_1.pdf]

Appendix A.

[redacted]

(a) TD

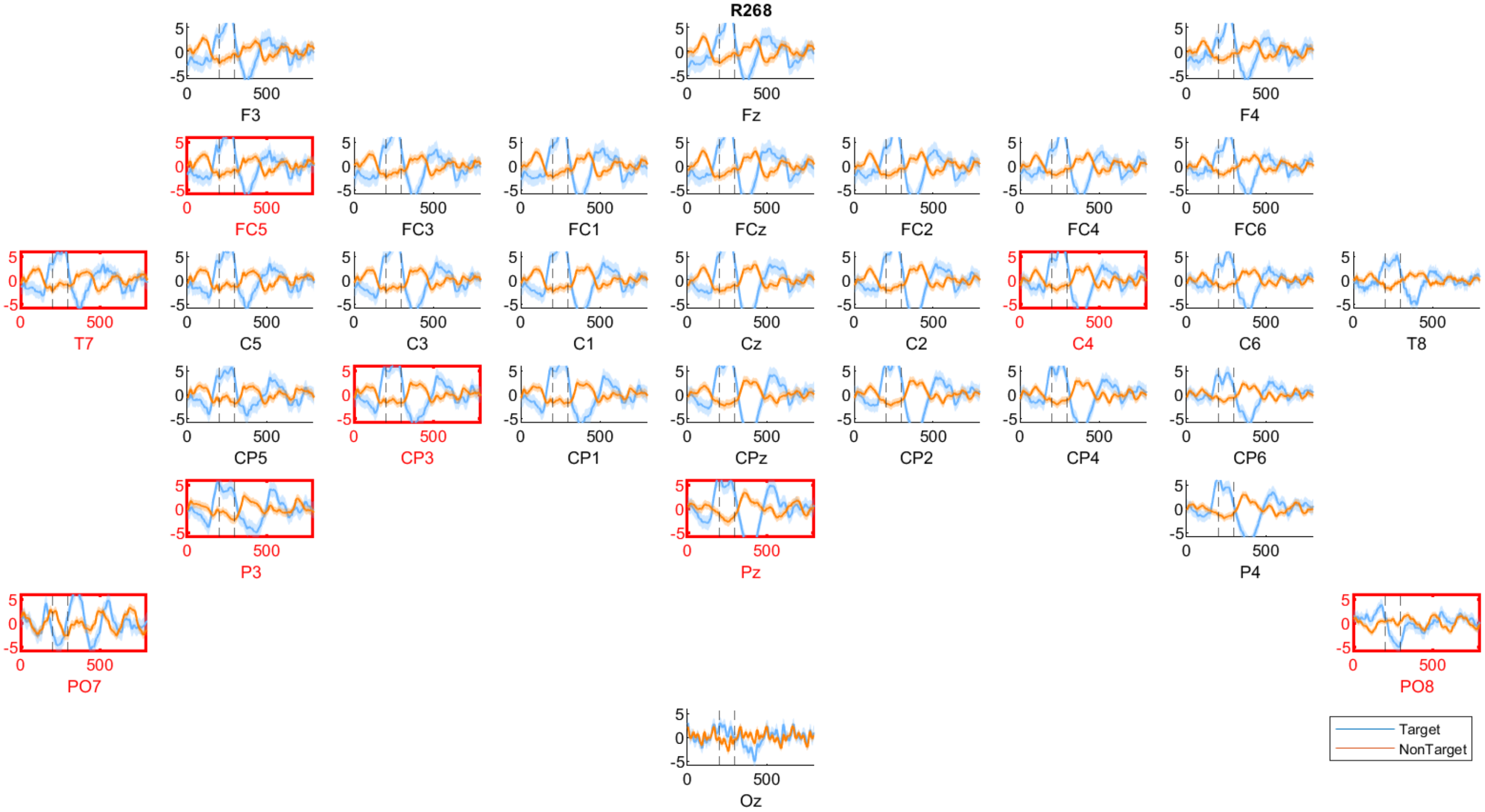

[redacted]

(b) Mild CP

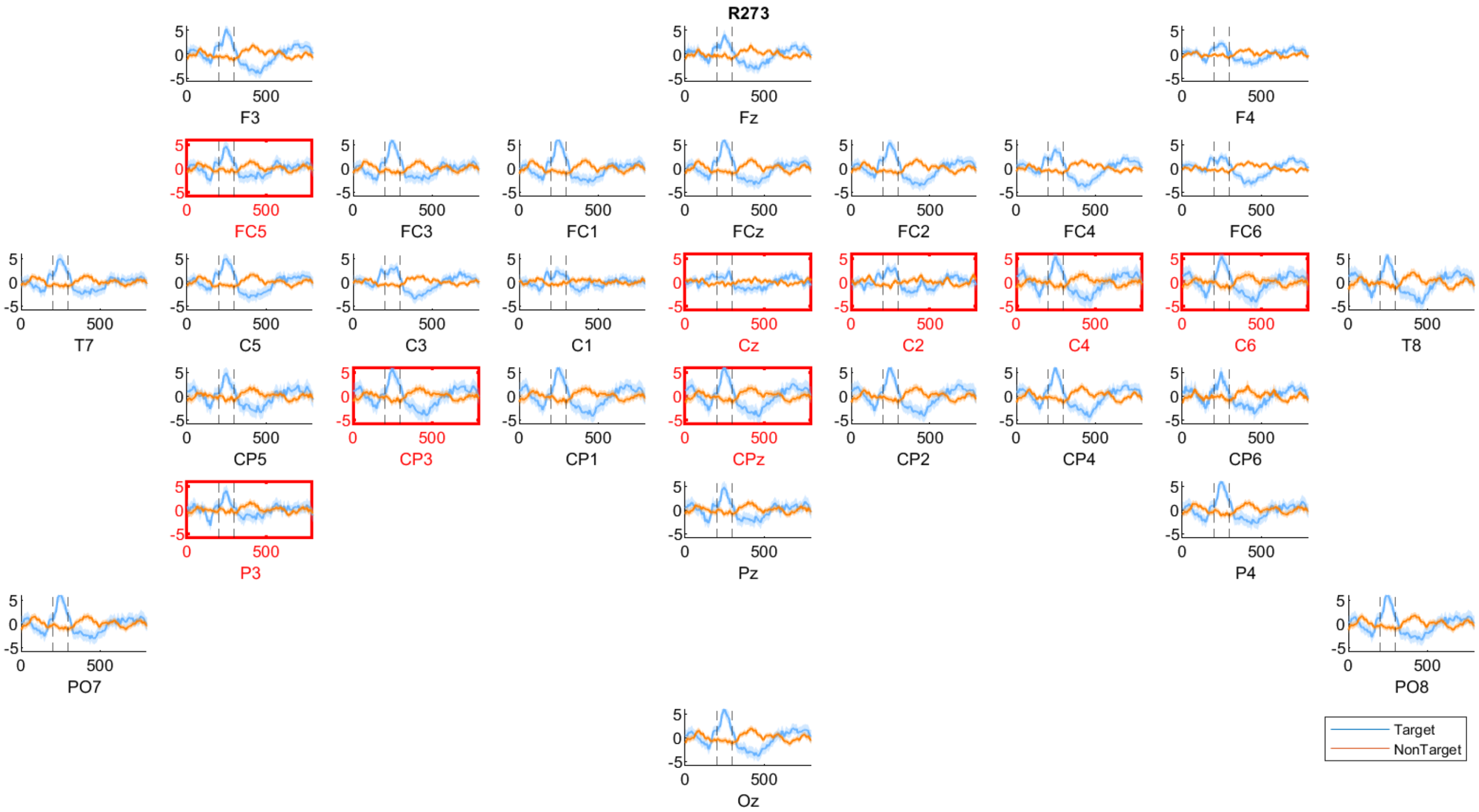

(c) Severe CP

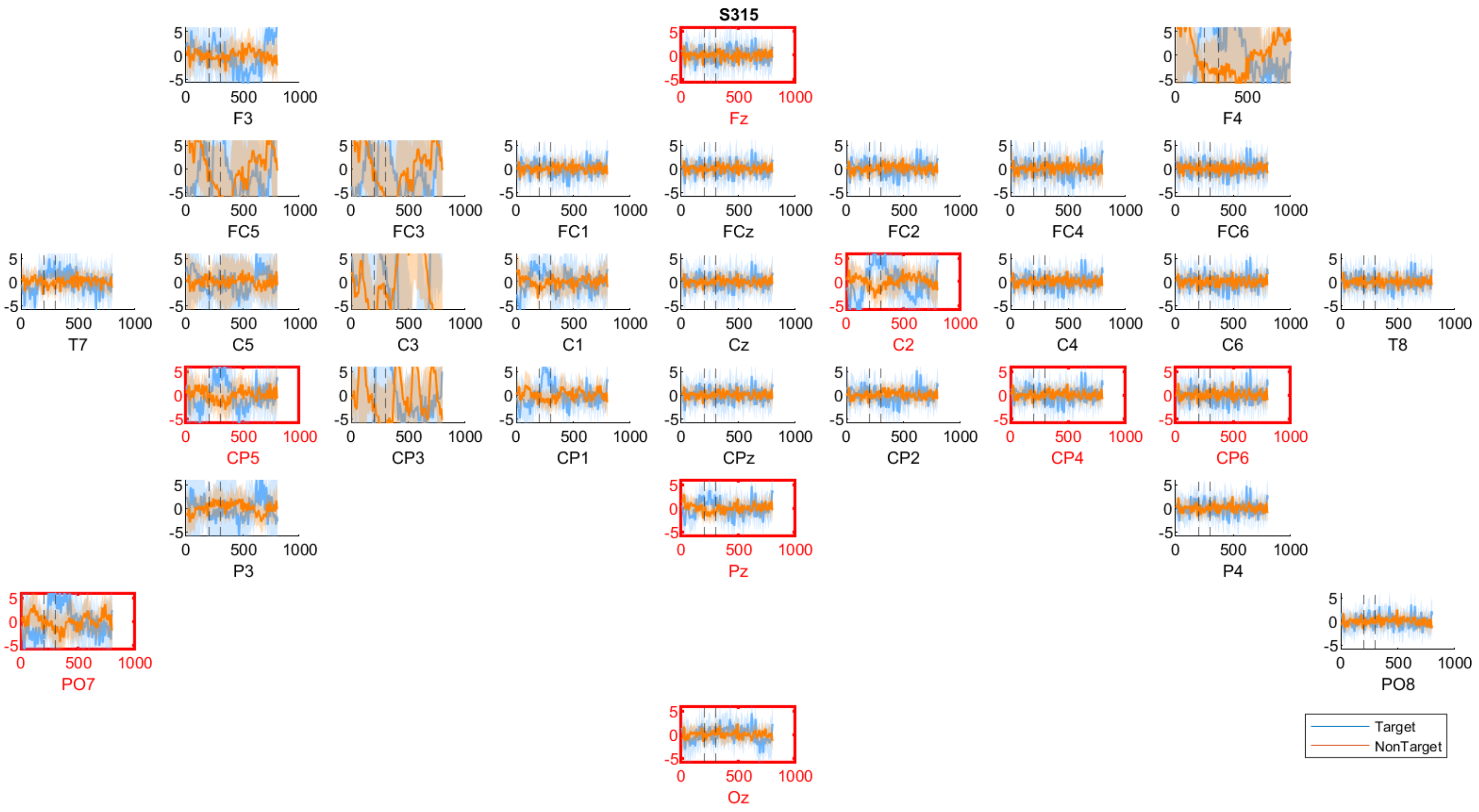

Figure Appendix A.1: Average target (blue) and non-target (orange) EEG of 30 trials for 800ms post stimulus. The 30 trials produce 300 target responses and 900 non-target responses. The shaded region represents the 95% confidence interval. The dotted reference lines mark t = 200 ms and t = 300 ms. Red boxes indicate the selected custom electrodes for the participant. (a) Example from the control group; (b) Example from the mild CP group; (c) Example from the severe CP group.
